# Synthetic TRuC receptors engaging the complete T cell receptor for potent anti-tumor response

**DOI:** 10.1038/s41467-019-10097-0

**Published:** 2019-05-07

**Authors:** Patrick A. Baeuerle, Jian Ding, Ekta Patel, Niko Thorausch, Holly Horton, Jessica Gierut, Irene Scarfo, Rashmi Choudhary, Olga Kiner, Janani Krishnamurthy, Bonnie Le, Anna Morath, G. Christian Baldeviano, Justin Quinn, Patrick Tavares, Qi Wei, Solly Weiler, Marcela V. Maus, Daniel Getts, Wolfgang W. Schamel, Robert Hofmeister

**Affiliations:** 1 TCR² Therapeutics, Inc., 100 Binney Street, Cambridge, MA 02142 USA; 2 Department of Immunology, Faculty of Biology, BIOSS Center for Biological Signalling Studies, CIBSS—Centre for Integrative Biological Signalling Studies and Centre for Chronic Immunodeficiency CCI, University of Freiburg, Schänzlestraβe 18, Freiburg, 79104 Germany; 3 Cellular Immunotherapy Program, Massachusetts General Hospital Cancer Center and Harvard Medical School, Bldg. 149 13th Street, Charlestown, MA USA

**Keywords:** Cell therapies, Cancer immunotherapy, Immunotherapy, T-cell receptor, Preclinical research

## Abstract

T cells expressing CD19-targeting chimeric antigen receptors (CARs) reveal high efficacy in the treatment of B cell malignancies. Here, we report that T cell receptor fusion constructs (TRuCs) comprising an antibody-based binding domain fused to T cell receptor (TCR) subunits can effectively reprogram an intact TCR complex to recognize tumor surface antigens. Unlike CARs, TRuCs become a functional component of the TCR complex. TRuC-T cells kill tumor cells as potently as second-generation CAR-T cells, but at significant lower cytokine release and despite the absence of an extra co-stimulatory domain. TRuC-T cells demonstrate potent anti-tumor activity in both liquid and solid tumor xenograft models. In several models, TRuC-T cells are more efficacious than respective CAR-T cells. TRuC-T cells are shown to engage the signaling capacity of the entire TCR complex in an HLA-independent manner.

## Abstract (web summary)

Supraphysiological T cell activation by chimeric antigen receptor (CAR) contributes to T cell exhaustion and adverse events in CAR T cell therapies. Here the authors engineer a synthetic antigen receptor that integrates into the endogenous TCR complex, preserving natural regulatory circuits and achieving improved performance in mouse tumor models.

## Introduction

The successful treatment of B cell malignancies with CD19-specific CAR-T cells has impressively demonstrated the therapeutic and curative potential of genetically engineered αβ T cells^1^. Complete response rates up to 90% have been reported for pediatric and adult patients with relapsed or refractory (r/r) acute B-lymphoblastic leukemia (ALL)^2,3^, and around 50% for r/r non-Hodgkin’s lymphoma patients (NHL)^4^. This high efficacy of CAR-T cells is surprising given the fact that CAR constructs only use the intracellular signaling domain of the CD3ζ chain in isolation from the five other subunits of the T cell receptor (TCR) complex.

The TCR is one of the body’s most complex receptors. The contribution and interplay of its six different receptor subunits to its very broad signaling activities in T cells is just beginning to emerge^5,6^. The TCR is composed of one TCRα and one TCRβ chain that together bind to the peptide-MHC ligand, and the signaling subunits, collectively called CD3: one dimer of CD3ε with CD3γ, one dimer of CD3ε with CD3δ, and one CD3ζ homodimer^7,8^. All subunits are type I membrane proteins and all but CD3ζ have extracellular immunoglobulin (Ig) domains.

The four different CD3 subunits have a total of ten immune receptor tyrosine-based activation motifs (ITAM) that together can accept up to 20 tyrosine phosphates upon activation^9^. Genetic mouse models have shown that the six ITAM motifs of the CD3ζ dimer are dispensable for establishing an intact immune system^10^, but not so a proline-rich sequence in the cytoplasmic tail of CD3ε, or a conformational change in CD3ε^11^. The activity of the TCR has been shown to be regulated by changes in the arrangement of its CD3 subunits that are stabilized by ligand binding to TCRαβ^12–14^, ligand-independent TCR nanoclustering^7,15,16^, and binding to cholesterol^17^.

In isolation from the TCR complex, CARs based on the intracellular domain of CD3ζ require for clinical activity insertion of the intracellular activation domains, e.g., as derived from the co-stimulatory receptors CD28 (28ζ) or CD137/4–1BB (BBζ). Such 28ζ and BBζ CARs are referred to as ‘second-generation’ CARs, and two respective CD19-specific CAR-T cell therapies (tisagenlecleucel and axicabtagene ciloleucel) have been approved in 2017 for the treatment of pediatric B cell precursor acute lymphoblastic leukemia and large B cell lymphoma, respectively^18–20^.

In the present study, we demonstrate that the same anti-CD19 scFv used for making 28ζ and BBζ CAR-T cells can be fused to the extracellular N-termini of any of the five other TCR subunits, resulting in the incorporation of the respective TCR fusion constructs (TRuCs) into the TCR complex. Resulting CD19-specific TRuC-T cells get activated by CD19-expressing target cells and can kill them as potently as CAR-T cells. In a mouse xenograft model using Raji cells, CD3ε-based TRuC-T cells showed better anti-tumor activity than 28ζ and BBζ CARs. We provide evidence that the enhanced in vivo activity of TRuC-T over CAR-T cells relates to profound differences in T cell signaling. TRuC-T cells employ the full signaling machinery of the TCR complex as opposed to CARs that use the limited signaling capacity of an isolated CD3ζ cytoplasmic tail, despite employing co-stimulatory domains derived from CD28 or 4–1BB.

## Results

### Design of TRuCs and lentiviral expression in human T cells

**Fig. 1:**
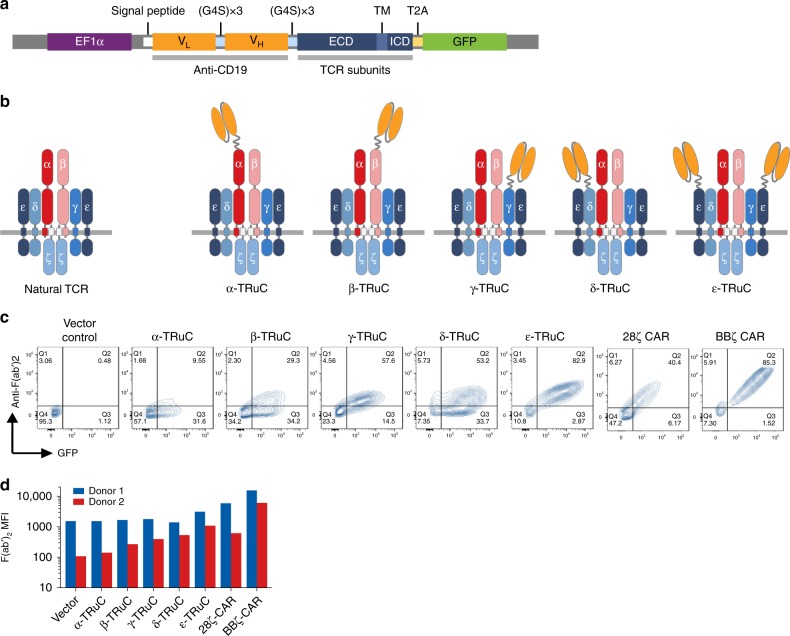
Design and surface expression of TRuCs. **a** Schematic diagram of the lentiviral vector expressing TRuC transgenes. The anti-CD19 scFv is tethered to full-length TCRα, TCRβ, CD3γ, CD3δ, or CD3ε TCR subunits via a flexible glycine serine linker. A T2A peptide enables co-expression of TRuCs and GFP under the EF1α promoter. **b** The natural TCR complex and five TRuC-containing TCR complexes. **c** Surface expression of TRuC variants or CARs in primary human T cells. T cells were transduced with lentiviral vectors encoding TRuCs or CARs and expanded in the presence of IL-2. The transduction efficiency was determined by the percentage of GFP-positive cells and the presence of the TRuC on the cell surface by anti-F(ab’)_2_ staining. **d** Quantification of the surface expression level of the five TRuCs and two CARs in two independent donors (*n* = 2). Representative data of two independent experiments are shown

We investigated whether the recombinant fusion of a single-chain variable fragment (scFv) to one of the TCR subunits can form a functional TCR complex and redirect T cells to kill tumor cells independent of HLA. The murine, anti-human CD19 scFv FMC63 was fused to the N-terminus of full-length human TCRα, TCRβ, CD3γ, CD3δ, or CD3ε. This scFv has been used to generate FDA-approved 28ζ and BBζ CAR constructs that showed high clinical activity in the treatment of leukemia and lymphoma patients^2,4,18,19^. We chose not to fuse FMC63 to CD3ζ since we have previously reported that enlargement of the short CD3ζ extracellular domain prevents integration of the resulting fusion protein into the TCR^21^, which was not the case for TCRβ^22^. Non-immunogenic (Gly_4_Ser)_3_ peptide linkers were employed for fusing the scFv to TCR subunits. To evaluate and control for transgene expression in different T cell preparations, green fluorescent protein (GFP) was co-expressed with all TRuCs and CARs using a T2A ‘cleavage’ site^23^. The key elements of the expression vectors are shown in Fig. 1a. Figure 1b depicts the five TCR fusion constructs (TRuCs) that were generated and characterized in more detail. Of note, the subunit stoichiometry of the TCR allows for the integration of up to two scFvs per receptor complex when fused to CD3ε, while fusion to the other subunits will result in one scFv per TCR.

The expression of all five TRuC variants, and of 28ζ CAR, BBζ CAR, and linked GFP, was assessed following lentiviral transduction of normal human donor T cells. Surface expression of the TRuC variants and of the two CARs on T cells was detected by flow cytometry using an anti-F(ab’)_2_ antibody recognizing the murine scFv frame work. The proportion of T cells with GFP expression was in the range of approximately 40–87%. However, the percentage of T cells with scFv expression on the cell surface greatly varied between 9 and 85%, which likely reflects differences in the efficiency of integration of TRuC variants into the TCR complex (Fig. 1c). The anti-F(ab’)_2_ mean fluorescence intensity of GFP-positive TRuC and CAR cells generated from two different donor T cells showed that the BBζ CAR was expressed highest on the T cell surface, followed by the 28ζ CAR and ε-TRuC. γ-TRuC, β-TRuC, α-TRuC, and δ-TRuC variants were expressed at similar levels, but to a lower extent than the ε-TRuC (Fig. 1d).

### Unlike CARs, TRuCs become an integral part of the TCR

Next, we investigated whether the five TRuC variants are incorporated into the human TCR and can trigger TCR-like signaling upon activation of T cells by harnessing the entire receptor complex. This would be a fundamental difference to CARs, which do not become an integral part of the TCR. CARs operate as stand-alone receptors utilizing only the cytoplasmic part of the CD3ζ chain of the entire TCR. To obtain larger numbers of cells for biochemical characterization of TCRs, TRuCs and CARs were stably expressed in the human Jurkat T cell line and homogeneous cell populations obtained by flow cytometric sorting of GFP^+^ cells. All TRuCs were expressed on the surface of Jurkat T cell line, with the δ-TRuC only showing low expression levels (Supplementary Fig. 1).

**Fig. 2:**
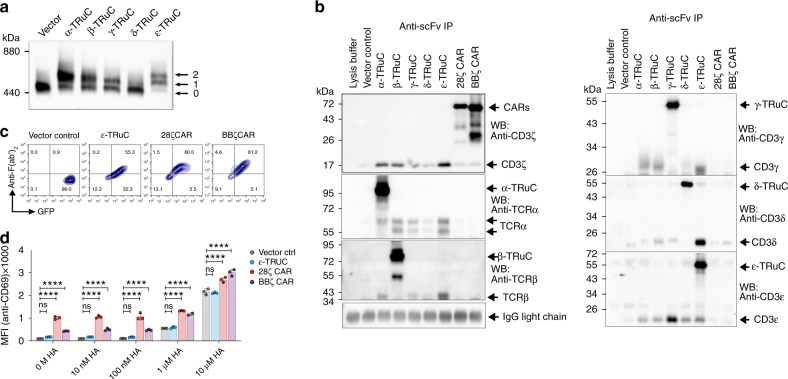
Incorporation of TRuCs in TCR complexes and functional analysis of endogenous TCR in Jurkat cells stably transduced with empty control vector or vectors encoding the indicated CAR or TRuC transgenes. The transgene expression was linked to GFP via a T2A sequence. **a** Cells were lysed and TCR complexes separated by Blue Native PAGE. After Western blot TCR complexes were stained using an anti-CD3ζ antibody. “0” denotes the natural TCR complex, “1” and “2” denote TCR complexes with one or two TRuCs, respectively. **b** Complex formation of TRuC variants with TCR subunits. Upon cell lysis, TRuCs and CARs were immunopurified using the anti-F(ab’)_2_ antibody, and then separated by reducing SDS-PAGE. As a control, the procedure was also applied to the lysis buffer alone. (Co)-purified proteins were detected using the described antibodies by western blot. Data in **a** and **b** show representative results of three experiments. **c** Transduced cells were stained with an anti-CD3ε antibody and analyzed by flow cytometry. **d** The effect of ε-TRuC on activation of an influenza HA-specific TCR. Jurkat T cells expressing the HA1.7 TCR (Jurkat cell line CH7C17) were transduced with a control vector (black) or vectors encoding for the ε-TRuC (light blue), the 28ζ (red) or BBζ CAR (purple). Cells were co-cultured with DapDR1-ICAM1 antigen-presenting cells loaded with different amounts of the HA306–318 peptide. After 6 h, upregulation of the activation marker CD69 was quantified by flow cytometry. The graphs in **c** and **d** show representative data of two independent experiments. Data were analyzed using a parametric two-way ANOVA test, followed by Dunnett’s multiple comparison test to compare means of the indicated sample to the control sample (Vector Control) at each HA concentration. ****Adjusted-*P* ≤ 0.0001. Error bars depict standard deviation. Samples were measured in triplicates

To demonstrate integration of TRuCs into the natural TCR, Jurkat cells were solubilized with the detergent digitonin, which keeps TCR complexes intact^7^, and TCRs purified as described^24^. T cell receptor complexes were then analyzed by Blue Native polyacrylamide gel electrophoresis^25^. In western blots using an anti-CD3ζ antibody, the native TCR complex was displayed as a discrete 450-kDa band in a non-denaturing gel (Fig. 2a)^7,26^. When α-, β-, or γ-TRuCs were expressed in Jurkat cells, additional, more slowly migrating TCR complexes appeared, consistent with the integration of TRuCs adding a 25-kDa scFv to the size of the TCR. Jurkat T cells expressing the ε-TRuC showed two slower migrating TCR complexes, in accordance with TCRs that harbor either one or two integrated ε-TRuCs (Fig. 2a). ε-TRuC integration appeared to be very effective because most of the native TCR complexes were converted in TRuC-containing receptor complexes. Quantification of the bands is shown in Supplementary Fig. 2. In Jurkat cells expressing the δ-TRuC, where only small amounts of TRuC can be found on the cell surface (Supplementary Fig. 2), a slower migrating TCR complex was barely visible.

Integration of TRuCs and CARs into the TCR complex was further analyzed by immunoprecipitation. TRuC and CAR complexes in Jurkat cell lines were immunoprecipitated with an anti-mouse F(ab’)_2_ antibody, separated by reducing SDS-PAGE, and analyzed by western blot for the presence of TRuCs, CARs, and endogenous TCR subunits. On the western blot, all immunoprecipitated TRuC and CAR proteins showed the expected molecular size (Fig. 2b). In addition, α-, β-, γ-, δ-, and ε-TRuCs all co-immunoprecipitated the six TCR subunits supporting the notion that all TRuCs were incorporated into the TCR. Only low amounts of endogenous TCR subunits were co-purified with the δ-TRuC. These results were in line with its low surface expression (Supplementary Fig. 2) and native gel electrophoresis data (Fig. 2a). The β-TRuC and the two CARs showed small amounts of additional smaller, presumably degraded species. Of note, the smaller 55 kDa form of the β-TRuC was not incorporated into the TCR. The ε-TRuC co-immunoprecipitated endogenous CD3ε, consistent with the presence of two CD3ε subunits in the TCR complex^7,8^. In contrast, the 28ζ or BBζ CARs did not co-immunoprecipitate any endogenous TCR subunit indicating that they were not associated with the endogenous TCR complex of Jurkat cells (Fig. 2b, and Supplementary Fig. 3).

The dependence of ε-TRuC surface expression on the presence of the TCR was further explored after lentiviral transduction of the TCR-negative multiple myeloma cell line RPMI-8226. While surface expression of the ε-TRuC was seen in Jurkat T cells, it was barely detected on the surface of the B cell line (Supplementary Fig. 4). In contrast, the 28ζ and BBζ CARs were expressed equally well on the surface of Jurkat cells and the multiple myeloma cell line RPMI-8226. These findings are consistent with the surface expression of CARs independent of the TCR.

### No interference of TRuC with pMHC-mediated T cell response

We studied whether the integration of TRuCs into the TCR interferes with T cell activation by a virus-specific TCR. To this end, we used the Jurkat cell line CH7C17 expressing the influenza-specific TCR HA1.7, which recognizes the hemagglutinin HA307–319 peptide in combination with HLA-DR1^27^. CH7C17 Jurkat cells transduced with vectors encoding the ε-TRuC, 28ζ CAR, or BBζ CAR expressed the respective transgenes on the cell surface detected with the anti-F(ab’)_2_ antibody (Fig. 2c). In the absence of peptide/HLA stimulation, the mere expression of CARs, but not of ε-TRuC resulted in a higher expression of T cell activation marker CD69 compared to the vector control (Fig. 2d). Upon stimulation of the HA1.7 TCR with influenza peptide/HLA-presenting antigen-presenting cells (APCs) for 6 h, CH7C17 control cells and cells transduced with the ε-TRuC upregulated of CD69 in a peptide dose-dependent manner. The extent of CD69 expression was not significantly different between ε-TRuC expressing and parental CH7C17 Jurkat cells suggesting that TRuCs do not impair normal TCR function. Of note, in our experiments, CARs induced a higher basic T cell activation, which could be the consequence of strong tonic signaling^28,29^. All TRuCs and CARs in Jurkat cells were functional, since co-culture with CD19-expressing tumor cells led to upregulation of CD69 in the transduced Jurkat cells (Supplementary Fig. 5).

### TRuC binding activates T cells to kill target cells

**Fig. 3:**
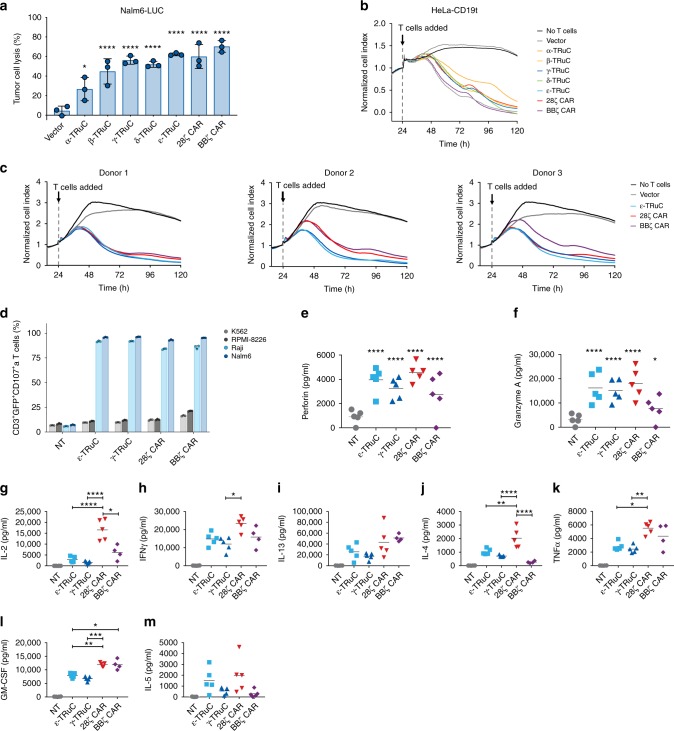
Characterization of TRuC-T cell cytotoxicity and cytokine production in vitro. **a** Tumor cell lysis of luciferase-expressing Nalm6 (Nalm6-LUC) tumor cells after 24 h at a T cell-to-target ratio of 1:1. Data represent results from three independent experiments. **b** Kinetics of lysis of HeLa cells expressing a truncated form CD19 (HeLa-CD19t) lacking the cytoplasmic domain. T cells and target cells were co-cultured at a ratio of 1:3. HeLa-CD19t lysis was measured in an impedance-based real-time cytotoxicity assay. Representative data of three experiments are shown. **c** Impact of donor variability on target killing of TRuC and CAR-T cells was assessed as in **b**. T cells derived from three different blood donors were transduced with TRuC or CAR variants and co-cultured at an effector-to-target ratio of 1:1. **d** CD107a degranulation by TRuC-T and CAR-T cells upon exposure to CD19-negative or CD19-positive tumor cells. Engineered and non-transduced (NT) T cells were co-cultured with the K562 (light grey), RPMI-8226 (dark gray), Raji (light blue), or Nalm6 (dark blue) at an effector-to-target ratio of 10:1. Error bars are standard deviation. Data represent results from two independent experiments. Samples in **a**–**d** were measured in triplicates. **e**–**m** Secretion of cytotoxic proteins or cytokines by non-transduced T cells (grey), ε-TRuC (light blue), γ-TRuC (dark blue), 28ζ CAR (red), or BBζ CAR-T cells (purple) following 24-hour co-culture with Nalm6 (1:1 effector-to-target ratio). After incubation, culture supernatants were collected and each analyte was quantified using a Luminex-based immunoassay. Data points represent individual donors (*n* = 5). Bars indicate group means. Statistical analysis was performed on log-transformed data using parametric one-way ANOVA followed by Dunnett’s (**e**–**f**) or Tukey’s (**g**–**m**) multiple comparison test comparing all groups to the non-transduced control group (**e**–**f**) or all groups against each other (**g**–**m**). Overall *P*-value was <0.001 for (**e**–**m**); * Adjusted-*P* ≤ 0.05, ** Adjusted-*P* ≤ 0.01, *** Adjusted-*P* ≤ 0.001, **** Adjusted-*P* ≤ 0.0001

We next tested whether TRuCs can activate the cytolytic program of T cells and how the cytolytic potential of TRuC-T cells compares to that of CAR-T cells. Redirected lysis by TRuC-T and CAR-T cells was assessed by two approaches: one was by percent lysis of luciferase-expressing Nalm6 (Nalm6-LUC) cells after 24 h (Fig. 3a); the other was by means of an impedance-based assay using CD19-expressing HeLa cells to study the kinetics of cell lysis (Fig. 3b, c). In both assays, low T cell-to-target cell ratios were used; based on the T cell transduction efficiency of ~25%, the ratio of TRuC-T or CAR-T to tumor cells was 1:4 and 1:12 in the luciferase endpoint and kill kinetics assay, respectively. Such low T cell-to-target cell ratios require serial target cell lysis by T cells in order to reach complete lysis.

In the Nalm6-LUC assay, all TRuC-T cells revealed significant target cell lysis above background of vector-transduced control T cells (Fig. 3a). ε-TRuC- and γ-TRuC-T cells showed the highest activity and were on par with 28ζ and BBζ CAR-T cells. α-, β-, and δ-TRuC-T cells showed a lower degree of Nalm6-LUC cell lysis after 24 h (Fig. 3a). In the impedance-based kill assay, we observed some variability in killing kinetics; however, all five variants of TRuC-T cells and the two CAR-T cell variants almost completely killed target cells after 120 h (Fig. 3b). The ε- and γ-TRuCs were selected to investigate the impact of donor variability on tumor cell lysis. As shown in Fig. 3c, the source of human donor T cells only slightly influenced the killing kinetics of ε- and γ-TRuC-T cells as well as of 28ζ and BBζ CAR-T cells.

Target cell lysis by TRuC-T and CAR-T cells correlated with the appearance of degranulation marker CD107a on the T cell surface (Fig. 3d). Between 80 and 90% of the transduced T cells presented CD107a-specific staining on their surface with no statistical difference between ε- and γ-TRuC T cells and 28ζ and BBζ CAR-T cells. Importantly, degranulation of T cells was only observed upon co-culture with the CD19-positive cell lines Raji and Nalm6, but not with the CD19-negative cell lines K562 and RPMI-8226, highlighting the antigen-specificity of target cell lysis. The levels of granzyme A and perforin, two key mediators of target cell lysis, were significantly increased after a 24-hours lytic reaction in the co-culture medium over the control, and reached similar levels for TRuC-T and CAR-T cells (Fig. 3e, f).

While target cell lysis, surface expression of CD107a, and release of granzyme A and perforin into the medium were similar between TRuC-T and CAR-T cells, both ε- and γ-TRuC-T cells produced upon target engagement less cytokines than the two CAR-T cell variants (Fig. 3f–m). Specifically, levels of IL-2, IFNγ, IL-4, TNFα, and GM-CSF, as measured after 24 h co-culture with NALM6 cells, were significantly higher in co-cultures with 28ζ CAR-T cells than with ε- and γ-TRuC-T cells. Cytokine release by BBζ CAR-T cells was more variable but also produced significantly higher GM-CSF levels after 24 h than the two TRuC-T cell variants.

### TRuC and CAR-T cells signal differently

**Fig. 4:**
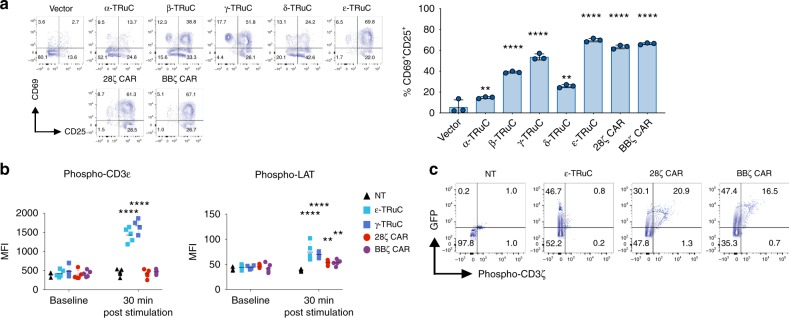
TRuC-T and CAR-T cell activation and signaling upon binding to CD19-positive target cells. **a** T cells were co-cultured overnight in the presence of Nalm6 tumor cells. T cell activation markers CD69 and CD25 were analyzed by flow cytometry. The graph depicts the percentage of CD69/CD25-double positive cells (mean ± SD of triplicates). Statistical analysis was performed on log-transformed data using parametric one-way ANOVA followed by Dunnett’s multiple comparison test comparing means of all groups to the vector control group. Representative data of three independent experiments are shown. **b** Phosphorylation of CD3ε and LAT in Non-transduced T cells (black), ε-TRuC (light blue), γ-TRuC (dark blue), 28ζ CAR (red), or BBζ CAR (purple) T cells upon co-culture with CD19-positive Raji cells at a 10:1 effector-to-target ratio for 30 min. Protein phosphorylation in cell lysates was analyzed using a Luminex-based cell signaling assay. Data points represent values for individual donors (*n* = 5) and bars indicate group means. Representative data of two experiments are shown. *P*-values were determined by two-way ANOVA for repeated measures followed by Dunnett’s multiple comparison test comparing all group means against non-transduced control group. Overall *P*-value < 0.001 for **a** and **b**; ** Adjusted-*P* ≤ 0.01, *** Adjusted-*P* ≤ 0.001, **** Adjusted-*P* ≤ 0.0001. **c** Representative plots showing phosphorylation of CD3ζ after 5 days expansion in the presence of IL-2 and anti-CD3/anti-CD28-coupled Dynabeads. CD3ζ phosphorylation in GFP-positive T cells was analyzed by flow cytometry. Graph shows representative data of two independent experiments

Because TRuC and CAR-T cells engage different TCR subunits to trigger the signaling cascade, we sought to explore whether there were differences in the quality of T cell activation between both engineered receptor types. Primary T cells transduced with CD19-specific TRuC or CAR variants were co-cultured with Nalm6 target cells overnight. In a subsequent flow cytometric analysis we determined the surface expression of the T cell activation markers CD69 and CD25 on GFP^+^ cells. T cells expressing the ε-TRuC and 28ζ and BBζ CARs showed a 60–66% conversion into an activated CD69^+^/CD25^+^ phenotype upon exposure to CD19-positive target cells, while conversion by the γ-TRuC was ~50% (Fig. 4a). T cells expressing α-, β-, or δ-TRuCs showed less expression of CD69, but relatively more expression of CD25.

A very early TCR signaling event is tyrosine phosphorylation of CD3ε^17,30^, which can be detected in T cells by intracellular anti-phospho-CD3ε staining. T cells expressing either ε- and γ-TRuCs strongly induced phosphorylation of CD3ε upon stimulation with CD19-positive target cells (Fig. 4b, left). In contrast, the CD3ε phosphorylation was not observed in T cells expressing the 28ζ or BBζ CARs. Likewise, we noticed increased phosphorylation of LAT, which is a signal amplification and diversification hub residing outside the TCR^31^ in ε- and γ-TRuC-transduced T cells compared to 28ζ and BBζ CAR-T cells. In the latter, LAT phosphorylation was only marginally increased upon stimulation (Fig. 4b, right).

The quaternary structure of TCRs keeps T cells in an auto-inhibited state, such that cytoplasmic tyrosine residues of CD3ζ and other CD3 subunits cannot be accessed by kinases in the absence of antigen, an important mechanism to keep resting T cells inactive^5,12,17^. To test whether the ε-TRuC containing TCR also adopts an inactive state in the absence of antigen, we analyzed CD19 ε-TRuC- or CAR-transduced T cells with an anti-phospho-CD3ζ antibody. ε-TRUC-T cells did not show a phosphorylated form of CD3ζ, whereas both CAR-T cell variants had significant levels of phosphorylated CD3ζ indicative of tonic signaling (Fig. 4c). This is in line with the constitutive CD69 expression seen in CAR-transduced Jurkat cells (Fig. 4d). In summary, the phosphoprotein analyses for CD3ε, CD3ζ, and LAT suggest that TRuC-T and CAR-T cells differ in the quality of intracellular signaling events.

Qualitative differences in signaling between TRuC- and CAR-T cells were also evident from Nanostring analysis. ε-TRuC-T cells, 28ζ CAR-T and BBζ CAR-T cells, and non-transduced T cells derived from three human donors were stimulated for 4 h with CD19-positive Raji B cells and mRNA for 594 immune related genes analyzed. While there was a considerable overlap in up and downregulated genes among the CAR and TRuC groups, a larger number of genes was uniquely up and downregulated in ε-TRuC-T cells compared to CAR-T cells that await further analysis (Supplementary Fig. 6).

### TRuC-T cells show enhanced activity in liquid tumor models

To determine how the integration of TRuCs into the TCR and the associated differences in early signaling events translated into anti-tumor response of TRuC-T cells, we established human leukemia and lymphoma mouse xenograft models for the side-by-side comparison of TRuC-T and CAR-T cells. To minimize potentially confounding effects and strengthen comparability, in all studies lentiviral vectors with the same EF1α promoter driving the expression of TRuC and CAR constructs and the same anti-CD19 scFv (FMC63) for target cell binding were used. T cells were transduced, activated, and expanded under identical conditions and a comparable number of TRuC-T or CAR-T cells injected into mice.

**Fig. 5:**
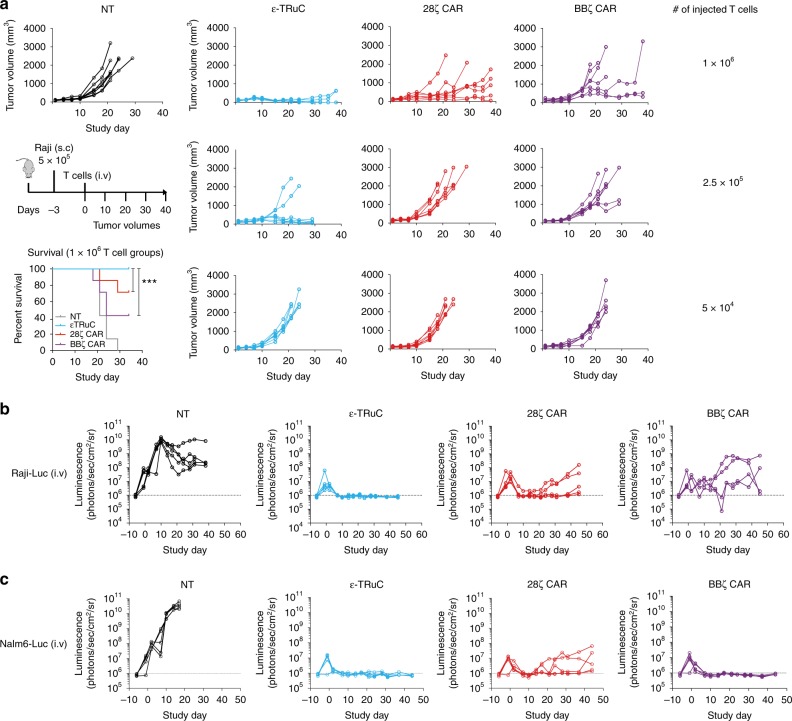
Anti-tumor activity of TRuC-T and CAR-T cells in lymphoma and leukemia tumor NSG xenograft models. **a** Raji-LUC tumor growth curves for individual mice upon treatment with CD19 ε-TRuC-T (light blue), 28ζ CAR-T (red), or BBζ CAR-T (purple) cells along with non-transduced (NT) control T cells (black). 5 × 10^5^ Raji tumor cells were subcutaneously injected in the flanks of NSG mice (*n* = 7 per group). Mice were treated with a single injection of the indicated T cell dose 3 days after injection of tumor cells, survival and tumor size was analyzed over 38 days. Survival curves were analyzed using the Log-rank (Mantel-Cox) test (***P < 0.0001). Data represent results from two independent experiments. **b** Growth curves of systemic Raji-LUC tumors upon treatment with CD19 ε-TRuC-T, CD19 28ζ CAR-T, CD19 BBζ CAR-T cells, or non-transduced (NT) control T cells. NSG mice (*n* = 5 per group) were injected with 5 × 10^5^ Raji-LUC cells into the tail vein of mice 5 days prior to treatment with 1 × 10^7^ non-transduced (NT) or engineered T cells. Tumor growth was monitored by bioluminescence imaging. Results from one experiment are shown. **c** Efficacy of CD19 ε-TRuC-T, CD19 28ζ CAR-T, CD19 BBζ CAR-T cells against disseminated Nalm6-LUC tumor cells. NT, non-transduced control T cells. NSG mice (*n* = 5 per group) were intravenously injected with 5 × 10^5^ Nalm6-LUC cells. Five days later, mice were treated with a single dose of 5 × 10^6^ non-transduced (NT) or engineered T cells. Tumor cell load in mice was monitored by bioluminescence imaging. Representative data from two independent experiments are shown

In a first in vivo study, NSG mice were subcutaneously inoculated with the CD19-positive Raji Burkitt lymphoma cell line. Once tumors were established, mice were treated with ε-TRuC-T, 28ζ CAR-T, or BBζ CAR-T cells by a single intravenous injection. At the highest T cell dose of 1 × 10^6^ cells, in five of seven mice no tumors could be detected for mice treated with ε-TRuC-T cells at the end of the study, while tumor escapes, some of them early, were observed in five of seven mice treated with 28ζ CAR-T or BBζ CAR-T cells (Fig. 5a). While outgrowth of subcutaneous Raji tumors was not affected by the lowest dose of TRuC-T or CAR-T cells (50,000 T cells), the next higher dose of 250,000 T cells still revealed anti-tumor activity for ε-TRuC-T cells, but not for 28ζ and BBζ CAR-T cells. In five of seven mice, the tumor outgrowth was prevented by the ε-TRuC-T cells. Survival was significantly different between ε-TRuC-T cell and BBζ and 28ζ CAR-T cells. Of note, in contrast to cell culture experiments, ε-TRuC-T cells appear to have higher and more sustained in vivo activity against localized tumor growth than the two CAR-T cell variants.

We next assessed the efficacy of TRuC-T cells in a disseminated Raji model, which was established by intravenous injection of firefly luciferase-expressing Raji cells into NSG mice. Six days later, ε-TRuC-T cells, 28ζ or BBζ CAR-T cells (10^7^ total T cells) were injected intravenously. ε-TRuC-T cell treatment led to an effective clearance of systemic Raji cancer cells in all mice treated (Fig. 5b). As observed for the subcutaneous RAJI model, reduced anti-tumor activity compared to TRuC-T cells was seen after injection of 28ζ CAR-T and BBζ CAR-T cells. After an initial response to CAR-T cells, tumor cells reappeared in the blood of the majority of treated mice.

To demonstrate that ε-TRuC-T cells can also show anti-tumor activity against tumor cells that express very low levels of co-stimulatory molecules, we used Nalm6-LUC cells to establish a disseminated leukemia model in NSG mice. Nalm6 cells express no CD80 and only low levels of CD86, ICOS, and 4–1BB (Supplementary Fig. 7). Mice were treated with the same batches of transduced T cells that were used in the systemic Raji model shown in Fig. 5b. Treatment with ε-TRuC-T cells resulted in rapid and robust tumor clearance of Nalm6-LUC leukemia in all four mice treated (Fig. 5c). In contrast, leukemia recurred in three of four mice treated with 28ζ CAR-T cells, but not in mice treated with BBζ CAR-T cells. These results indicate that the anti-tumor activity of TRuC-T cells is not dependent on the expression of ligands for co-stimulatory molecules on target cells.

### TRuC-T cells are also efficacious against solid tumors

To investigate a broader utility of TRuC-T cells, we generated TRuC-T cells using antibody domains specific for the multiple myeloma target B Cell Maturation Antigen (BCMA) and for the glioblastoma multiforme (GBM) target interleukin-13 receptor α2 (IL-13Rα2).

**Fig. 6:**
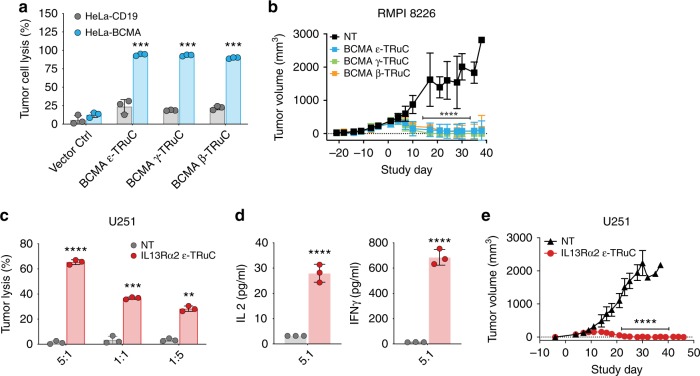
Cytotoxic activity of BCMA- and IL-13Rα2-specific TRuC-T cells. **a** BCMA-specific TRuCs were generated by fusing a BCMA-specific scFv to CD3ε, CD3γ or TCRβ subunits. Lysis of HeLa cells expressing BCMA (light blue) or, as control, expressing CD19 (grey) by the respective TRuC-T cells was measured at an effector-to-target cell ratio of 1:1. Graphs depict data from two independent experiments measured in triplicates. **b** Analysis of BCMA-specific ε-TRuC-T (light blue), γ-TRuC-T (green), β-TRuC-T (orange) or non-transduced control T cells (black) for anti-tumor activity in a RPMI-8226 NSG mouse model for multiple myeloma. Data from one experiment are shown. **c** An IL-13Rα2-specific ε-TRuC was generated by fusing a single-domain antibody (VHH) to CD3ε. IL-13Rα2-specific ε-TRuC-T (red) or non-tranduced (NT) T cells (black) were tested for in vitro lysis of U251 glioblastoma cells at various effector-to-target cell ratios. **d** Release of IL-2 and IFN-γ by IL-13Rα2-specific TRuC-T cells activated by U251 target cells. Figures **c** and **d** show data from four independent experiments measured in triplicates. **e** Activity of IL-13Rα2-specific ε-TRuC-T cells against subcutaneous U251 tumors in a NSG mouse model. Results from one experiment are shown. Data were log-transformed and analyzed using either two-way ANOVA followed by Dunnett’s multiple comparison test comparing all group means against the control group (vector control group for **a** or NT control group for **b**, **c**, and **e**) or two-tailed Student’s *t*-test. Overall *P*-value ≤ 0.0001 for **a**, **b**, **c**, and **e**. *** Adjusted-*P* ≤ 0.001; **** Adjusted-*P* ≤ 0.0001. Error bars in all graphs depict standard deviation

An anti-BCMA scFv was used to construct ε-, γ-, and β-TRuCs as described for CD19-specific TRuCs (see Fig. 1). All three resulting BCMA-specific TRuC-T cells effectively lysed in co-culture assays BCMA-expressing HeLa cells, but not HeLa cells expressing CD19 (Fig. 6a). In a NSG model using human multiple myeloma cell line RPMI-8226, a single dose of either ε-, γ-, and β-TRuC-T cells was sufficient to regress and control tumor growth for up to 40 days (Fig. 6b). T cells transduced with the control vector could not prevent outgrowth of tumors.

Finally, we investigated how effective TRuC-T cells are against a solid tumor target. A TRuC was made by fusing a single-domain antibody (VHH) specific for IL-13Rα2 to the CD3ε subunit. The resulting IL-13Rα2-specific ε-TRuC-T cells lysed target-positive U251 glioblastoma cells at various effector-to-target ratios (Fig. 6c) and caused release of IFN-γ and IL-2 by T cells (Fig. 6d). In a subcutaneous U251 NSG model for GBM, IL-13Rα2-specific ε-TRuC-T cells eliminated subcutaneous tumors and prevented re-growth of tumors for up to 48 days (Fig. 6e). In conclusion, functional TRuC-T cells can be made by fusing different antibody binding formats (e.g., scFv or VHH) thereby recognizing distinct tumor antigens.

## Discussion

We here show that the N-termini of TCRα, TCRβ, CD3γ, CD3δ, and CD3ε subunits of the TCR can be recombinantly fused using a linker sequence to an antigen-binding scFv or single-domain antibody, thereby providing the TCR and the engineered T cell with a new target specificity and the potential for HLA-independent target cell lysis. For an in-depth comparison of the TRuC approach with two established second-generation CAR-T cell designs, we selected the CD19 target. In a large set of experiments, TRuC-T cells were either superior or equivalent to CAR-T cells (Supplementary Table 1).

All five CD19-specific TRuCs were integrated into the TCR complex and could potently activate T cells upon interaction with CD19-expressing target cells. In contrast to a previous attempt that failed to make functional scFv fusion proteins with TCR subunits^32^, all our TRuC variants recognized target cells and could potently eliminate them.

Evidence for a functional integration of TRuCs into the TCR is manifold: (i) TRuCs were expressed on the T cell surface as shown by surface staining with an antibody against the fused scFv. This is indicative of their assembly into the TCR because isolated TCR subunits are not well transported to and expressed on the cell surface^33^. Observed differences in the surface expression of TRuCs may therefore relate to how efficiently they integrate into the TCR. (ii) Unlike CARs, TRuCs could co-immunoprecipitate all other TCR subunits. The ε-TRuC uniquely co-immunoprecipitated endogenous CD3ε, which is in line with the stoichiometry of the TCR containing two CD3ε subunits^33^. (iii) In a native electrophoretic analysis of the TCR complexes, we detected complexes larger than the endogenous “normal” TCR. These were TCRs including one TRuC or, in case of the ε-TRuC, two ε-TRuCs. (iv) Activation of TRuC-T cells caused tyrosine phosphorylation of CD3ε and CD3ζ, suggesting activation of the complete TCR complex.

In our in vitro experiments, the five TRuC variants showed discrete differences with respect to the activity of engineered T cells. Most active were ε- and γ-TRuCs followed by β- and α-TRuCs. Least active were T cells expressing the δ-TRuC. These differences may relate to how well TRuCs integrate into the TCR and thereby reach the T cell surface and, consequently, how well the integrated TRuCs can bind their antigen on target cells.

In signaling-active TCR microclusters, the membranes of T cell and target cell come into close contact because both peptide-MHC and TCR are rather small surface proteins. The close approximation of membranes results in exclusion of the large phosphatases CD45 and CD148 on T cells. Exclusion prevents constitutive dephosphorylation of CD3 subunits and thereby allows for strong signaling^34,35^. This might also be possible if TRuCs embedded in TCRs bind their respective surface target with a similar geometry and spacing as peptide/MHC complexes. It may also explain why TRuCs made by scFv fusion to TCRα and TCRβ had a somewhat lower activity. Their longer extracellular domains will increase the cleft of the cytolytic synapse, which may be less effective in exclusion of bulky phosphatases. On the other hand, cytolytic synapses formed by ε-TRuCs may have the highest stability of all TRuCs because two CD3ε subunits are present in the TCR that can engage in target antigen binding. Based on their highest activity, we have selected ε-TRuCs for making engineered T cells to be tested in clinical trials.

In the present experiments, we have compared CD19-specific TRuCs to two variants of CD19-specific CARs that have shown very high activity in clinical trials with leukemia and lymphoma patients^18,19^. We used the same anti-CD19 scFv for making TRuCs and CARs. TRuC surface expression was shown to be dependent on the association with a TCR in T cells, while CARs could be expressed on the cell surface in both T and non-T cells. Recently, “by chance” CAR expression on the surface of oncogenic B cells caused tumor relapse by masking the CD19 epitope on the B cells^36^. Since TRuC due to the lack of the other TCR subunits are not expressed on the surface of B cells, a TRuC-T cell therapy might be safer. Another concern might have been that TRuCs influence the ability of the TCR to recognize peptide/MHC. However, here we show that expression of an ε-TRuC did not alter the activity of T cells specific for the influenza virus derived HA peptide presented by HLA-DR1 on antigen-presenting cells.

The independence of CARs from expression and regulation of the TCR may explain an over-activation and exhaustion of T cells observed with CAR-T cells^28^. While the TCR controls T cell activation by autoregulatory mechanisms involving the transmembrane region of TCRβ^5,17,37^, CAR-T cells cannot benefit from this intricate regulation of the TCR complex. This may explain the presence of phospho-CD3ζ after long-term stimulation by CARs, and its absence in TRuC-T cells. Likewise, expression of CARs, but not of the ε-TRuC, caused CD69 upregulation in Jurkat cells in the absence of antigen stimulation.

It is evident from our signaling and gene expression analyses that although TRuCs and CARs activate basic T cell signaling pathways, there are also clear differences. The signaling events induced by TRuCs were consistent with activation of the entire TCR, e.g., the phosphorylation of CD3ε and CD3ζ, which was not seen with CARs. While there was a substantial overlap of newly regulated genes, TRuCs and CARs also showed significant differences upon T cell activation. Such differences may have consequences for the duration and expression level of immunomodulatory factors and ultimately the performance of engineered T cells. Dozens of differentially activated factors now await further analysis.

It is noteworthy that CD19-specific ε- and γ-TRuC-T cells released significantly less cytokines into the co-culture medium than CAR-T cells. The two CAR variants also showed significant differences between each other in that the 28ζ CAR showed the stronger cytokine response, which is consistent with previous reports^38^. Of note, the extent of cytokine release did not correlate with the cytotoxic potential of engineered T cells. It is conceivable that a reduced cytokine production by CD19 TRuC-T cells could result in a better safety profile in clinical trials where cytokine release syndrome is a frequently observed severe adverse event^39,40^. The TCR is known to not only mediate a broad and complex signaling in T cells but also to precisely control the timing of signaling events^41^. A reduced cytokine release could therefore be the result of a limited signal duration that is only possible with signaling through a complete TCR.

Importantly, in a Raji subcutaneous lymphoma xenograft mouse models, ε-TRuC-T cells demonstrated superior tumor killing compared to both CD28ζ and BBζ CAR-T cells. Control of the subcutaneous tumor required less TRuC-T cells than CAR-T cells, and tumor control by TRuC-T cells was more effective. This was unexpected given that the ε-TRuC- and CD28ζ and BBζ CAR-T cells used in the animal experiment were equally potent in in vitro cytotoxicity assays. It is possible that TRuC-T cell activation and signaling through the complete TCR allows for superior T cell performance against both localized tumors and disseminated disease. This could relate to better tumor penetration, longer persistence, and/or less exhaustion by the tumor microenvironment. Likewise, in a disseminated Raji model, TRuC-T cells were able to clear tumors and prevent recurrence. Of note, TRuC-T cells cleared tumor in a Nalm6 leukemia model, which is known for its low expression of co-stimulatory ligands CD80 and CD86. This suggests that the efficacy of TRuC-T cells is not dependent on the engagement of these co-activator molecules to drive anti-tumor activity. It is likely that other adaptor molecules that are recruited by other than the CD3ζ subunit contribute to the activation and anti-tumor response of TRuC-T cells^42^. Future in vivo studies are required to elucidate the basis for differences in the performance of TRuC-T and CAR-T cells.

Anti-tumor activity of TRuC-T cells in the multiple myeloma and GBM models suggests that TRuC-T cells are broadly applicable to a variety of hematological malignancies and solid tumors. Furthermore, we demonstrated that TRuC-T cells incorporating fusion constructs with scFv or sdAb binders trigger cytokine release, redirect T cells to kill tumor cells and clear tumors in mouse models. These findings further underpin the versatility of the TCR fusion constructs.

Despite the astounding efficacy of second-generation CD19-specific CAR-T cells in certain B cell malignancies, little if any activity was thus far observed with CAR-T cells in solid tumors^43,44^. This is attributed to shortcomings of target antigens and scFvs, issues with toxicity, tumor penetration, and exhaustion of CAR-T cells, and the impact of the immunosuppressive tumor microenvironment^45,46^. However, there are other T cell therapies that have shown impressive clinical activity in solid tumors after adoptive transfer. These are ex vivo expanded, tumor-infiltrating lymphocytes (TILs), and T cells engineered to express TCRα and TCRβ chains of predefined specificity^47^. Therapy with TILs has demonstrated large tumor eradication and long-term survival in melanoma patients^48^, and therapies with NY-ESO1-specific TCR-T cells gave objective response rates in melanoma and synovial sarcoma of >50%^1^. Notably, in contrast to CAR-T cells, such T cell therapies use T cells with a complete TCR complex for targeting cancer cells and for T cell signaling and activation. It is therefore conceivable that T cells require the more comprehensive and physiological signaling of a complete TCR complex to be active against solid tumors rather than signaling by an isolated CD3ζ subunit and co-stimulatory domains. Future clinical studies with TRuC-T cells specific for solid tumor antigens will investigate this possibility.

## Methods

### Lentiviral expression constructs

CD19-targeting TRuC variants were generated by tethering the FMC63 (AA 1–267, GenBank ID: HM852952.1) single-chain Fv (scFv) sequence to various TCR subunits via the flexible linker (GGGGS)x3 using gene synthesis. TCRα and β variable domain sequences were described by Yoshikai et al. and Lopez et al., respectively^49,50^. The constructs were cloned into the pCDH-EF1-MCS-T2A-copGFP expression plasmid from System Bioscience (SBI, Palo Alto, CA) using the XbaI and EcoRI restriction sites. TRuC expression was coupled with a GFP reporter gene (copGFP) via a 2A-like sequence (T2A). For the generation of target cell lines, full-length firefly luciferase or CD19 lacking the cytoplasmic domain were cloned into pCDH-EF1-MCS-T2A-Puro (SBI, Palo Alto, CA) using XbaI and EcoRI restriction sites.

### Lentiviral vector production

Lentivirus for the transduction of Jurkat cells was prepared as following. HEK293T cells were transfected with the respective constructs cloned into pCDH-EF1-MCS-T2A-copGFP (SBI, Palo Alto, CA) and the packaging plasmids pMD2.G (Cat #11259, Addgene, Cambridge, MA) and pCMVR8.74 (#22036, Addgene, Cambridge, MA) in HEK293T cells. For primary T cell transduction, 293TN cells (SBI, Palo Alto, CA) were transfected with the respective expression plasmids and the pPackH1 Packaging mix (SBI, Palo Alto, CA) using lipofectamine 3000 (ThermoFisher, Waltham, MA). Virus-containing supernatants were harvested and concentrated prior to transduction of cells.

### Cell lines

Jurkat E6, RPMI-8226, K562, Raji, Hela cell lines were purchased from ATCC, Manasas, VA. CH7C17 T cells, expressing the human HA1.7 TCR, and DapDR1-ICAM1 cells, expressing HLA-DR1, were a gift of Andres Alcover, Paris and Balbino Alarcon, Madrid. For immunoprecipitation experiments, CAR or TRuC-transduced Jurkat cells were sorted twice to enrich for the highest GPF-expressing Jurkat cells. The Nalm6 cells line was obtained from DSMZ, Germany. All cells were maintained in culture media recommended by the manufacturer. Nalm6 cells were modified to express firefly luciferase. Hela and K562 cell lines were transduced with lentivirus encoding truncated CD19. Upon transduction, stable cell lines were generated by puromycin selection (Corning, Bedford, MA).

### Primary human T cell activation, transduction, and expansion

Primary human T cells were isolated from leukapheresis product (Hemacare, Van Nuys, CA) by magnetic bead separation using anti-CD4 and anti-CD8 microbeads according to the manufacturer’s protocol (Miltenyi Biotech, Bergisch Gladbach, Germany). T cells were activated using anti-CD3/CD28 coupled Dynabeads (Life Technologies, Carlsbad, CA) at a 1:1 ratio and cultured in AimV (LifeTechnologies, Carlsbad, CA) with 5% human AB serum (Gemini bio, West Sacramento, CA) and 300IU/ml IL-2 (Peprotech Rocky Hill, NJ). T cell transduction was carried out using spin oculation at 100 × *g* for 100 min in presence of 5 μg/ml polybrene (Sigma, Natick, MA) and a MOI of 1 of the respective lentivirus. T cells were cultured for 8–10 days prior to use in functional assays.

### TRuC or CAR surface expression

TRuC or CAR expression on cells was analyzed by flow cytometry. Live Dead Aqua dye (Thermo Fisher, Waltham, MA) was used according to the manufacturer’s instructions to determine live cells. TRuC or CAR surface expression was detected with a goat anti-mouse F(ab’)_2_ biotin antibody (Invitrogen, Carlsbad, CA) followed by a secondary streptavidin-PE antibody (BD Biosciences, San Jose, CA). For T cell profiling the following antibodies were used: anti-CD3 (UCHT1), anti-CD8 (SK1), anti-CD4 (RPA-T4), and appropriate isotype controls (BD Biosciences, San Jose, CA). Samples were analyzed using the BD LSR Fortessa X-20 cell analyzer (BD Biosciences, San Jose, CA). Data analysis was performed with the FlowJo software (Treestar Inc, Ashland, OR).

### Luciferase activity-based cell lysis assay

Luciferase-expressing tumor cells were plated in triplicates in a 96-well plate at 5000 cells per well and T cells added at the desired effector-to-target (E:T) ratios. After 24-hour culture, 50% of the culture supernatant was removed. Cell viability was determined using the Bright-Glo™ Luciferase Assay System (Promega, Madison WI) according to the manufacturer’s protocol. Relative luminescence (RLU) was measured using the SpectraMax M5 plate reader (Molecular Devices, Sunnyvale, CA). The percentage of tumor lysis was calculated by the following formula: % tumor cell lysis = 100% × (1 – RLU (tumor cells + T cells)/RLU (tumor cells).

### Impedance-based kinetics cell lysis assay

Using the impedance-based xCELLigence system (ACEA Biosciences Inc, San Diego CA), the kinetics of tumor cell lysis was evaluated over 144 h. HeLa-CD19t tumor cells were plated in a 96-well, resistor-bottomed plate at 10,000 cells per well in triplicates. After 24 h, effector T cells were added to adjust the desired effector-to-target (E:T) ratios. The impedance was measured in 15-minute intervals. The impedance-based cell index for each well and timepoint was normalized with the cell index prior to the addition of T cells. Kinetics of tumor cell lysis is depicted as change in normalized cell index over time.

### CD107a degranulation assay

TRuC or CAR-T cells were co-cultured with one of the following target cells: Raji, RPMI-8226, K562 and Nalm6 cell lines. T cells and target cells were plated at an effector-to-target ratio of 1-to-1 in a 96-well U bottom plate. Anti-CD107a antibody (clone-H4A3) was added to the co-culture for 1 h at 37 °C, 5% CO_2_. Then, the protein transport inhibitor monensin was added per manufacturer’s instructions and cells incubated for additional 3 h. Subsequently, T cells were labelled with the following antibodies: anti-CD3, (clone UCHT1), anti-CD4 (RPA-T4), and anti-CD8 (SK1) (BD Biosciences, San Jose, CA). Samples were acquired using the BD LSR Fortessa X-20 cell analyzer (BD Biosciences, San Jose, CA) and data analyzed using the FlowJo software (Treestar Inc.).

### Luminex-based cytokine detection

The secretion of cytokines into co-culture supernatant was measured using the Luminex-based MILLIPLEX MAP Human CD8^+^ T Cell Magnetic Bead Panel Premixed 17 Plex—Immunology Multiplex Assay (MilliporeSigma, Billerica MA). The culture supernatant was collected after 24 h of co-culture and stored at −80 °C until sample analysis. The detection of cytokines was carried out per manufacturer’s instruction.

### TRuC or CAR-T cell activation marker analysis

TRuC-T and CAR-T cells were co-cultured overnight with CD19^+^ Nalm6-LUC target cells or CD19^−^ K562 target cells at 1:1 ratio in triplicates. Alternatively, CH7C17 cells were co-cultured with DapDR1-ICAM1 cells loaded with different amounts of the HA306–318 peptide. T cell activation markers were analyzed using anti-human CD25 (clone BC96) (eBioscience, San Diego, CA), anti-human CD69 (clone FN50) and anti-CD3 (clone UCHT1) (BD Biosciences, San Jose, CA).

### Immuno-purification and western blotting

The following antibodies and reagents were used for biochemical analysis: anti-TCRα (clone H-1, #sc-515719, Santa Cruz), anti-TCRβ (clone H-197, #sc-9101, Santa Cruz), anti-CD3γ (clone EPR4517, #3256–1, Epitomics), anti-CD3δ (clone F-1, #sc-137137, Santa Cruz), anti-CD3ε (clone M20, #sc-1127, Santa Cruz, and clone OKT3, #14–0037–82, Thermo Fisher), anti-CD3ζ (serum 449, described in Deswal et al.^51^), anti-HA tag (12CA5, #MA1–12429, Thermo Fisher), biotin-coupled anti-mouse IgG (Fab´)_2_ biotin (#31803, Thermo Fisher), horseradish peroxidase (HRPO)-coupled anti-mouse IgG (#32430, Thermo Fisher), HRPO-coupled anti-goat IgG (#31402, Thermo Fisher), and HRPO-coupled anti-rabbit IgG (#31460, Thermo Fisher). Paramagnetic streptavidin-coupled sepharose (#28–9857–99) and protein G-coupled sepharose (#17–0618–01) were from GE Healthcare and the protease inhibitor cocktail from Sigma.

3 × 10^7^ cells were lysed in 1 ml lysis buffer containing 20 mM Tris-HCl pH8, 137 mM NaCl, 2 mM EDTA, 10% glycerol, 1x protease inhibitor cocktail, 1 mM PMSF, 5 mM iodoacetamide, 0.5 mM sodium orthovanadate, 1 mM NaF, and 0.5% Brij96 for 30 min at 4 °C followed by 15 min centrifugation to pellet the nuclei and insoluble material. For the anti-CD3ε immunoprecipitation 250 μl cleared cell lysate was incubated with 5 μl 50% protein G sepharose slurry and 1 μg anti-CD3ε OKT3 or 1 μg anti-HA tag control antibody for 2 h at 4 °C. For the anti-F(ab’)_2_ immunoprecipitation, 400 µl cleared cell lysate was incubated with 6 μl paramagnetic streptavidin-coupled sepharose and 20 μg anti-scFv antibody for 2 h at 4 °C. After four washes, the immunoprecipitated material was separated by 10–15% reducing SDS-PAGE. The separated proteins were transferred to PVDF membranes by semi-dry transfer. After blocking with 5% milk in PBS containing 0.1% Tween-20 the membranes were incubated with antibodies against TCRα (1:1000), TCRβ (1:100), CD3γ (1:1000), CD3δ (1:100), CD3ε (1:1000), CD3ζ (1:1000) in PBS-T followed by incubation with HRPO-conjugated secondary antibodies (1:10000). Western blot signals were recorded using an Image Quant LAS 4000 Mini from GE Healthcare Life Sciences, Boston, MA.

### Blue Native PAGE analysis

Jurkat cells were treated with the protein tyrosine phosphatase inhibitor pervanadate to phosphorylate TCR tyrosine residues, and then lysed in 1% digitonin. Phosphorylated TCRs were immunopurified from cellular lysates using the anti-phosphotyrosine antibody 4G10. The immunopurified TCR complexes were eluted from the beads using 100 mM phenylphosphate and 0.3% digitonin and separated under native conditions using BN-PAGE (4–8%). TCR complexes were detected by western blotting using the anti-CD3ζ antibody 449. We had shown earlier that phosphorylation of the TCR did not change its stoichiometry^7^.

### Phosphoprotein analysis

To measure baseline phosphorylation of CD3ζ, T cells were stimulated at day 0 with Dynabeads at a 3:1 ratio. Following the transduction with lentivirus on day 1, T cells were expanded for 5 days in presence of 20 U/ml IL-2. Upon harvest, 10^6^ cells were fixed with 4% paraformaldehyde and permeabilized using BD Perm Buffer III per manufacturer’s recommendation. Anti-phospho-CD3ζ (Clone, K25–407.69) used for intracellular staining was from BD biosciences. For CD19-induced CD3ε and LAT phosphorylation, transduced T cells from up to five individual normal donors were expanded 9 days in the presence of Dynabeads and IL-2. Then, cells were harvested and starved overnight in Aim V media containing 0.5% human AB serum. For T cell stimulation, T cells and Raji cells were co-cultured at an effector-to-target ratio of 10-to-1 for 30 min. Phosphorylation of the TCR subunits was measured using the Milliplex MAP 7-plex Human T-cell Receptor magnetic bead kit (MilliporeSigma, Billerica MA). Sample acquisition and analysis was performed using MAGPIX Luminex xMAP Technology according to manufacturer’s instructions (MilliporeSigma, Billerica MA).

### Gene expression analysis

Twenty million (2 × 10^7^) ε-TRuC-T, CD28ζ-T, and BBζ CAR-T cells were co-cultured with 2 × 10^7^ Raji cells (effector-to-target ratio of 1:1) for 4 h. Raji cells were depleted using CD19 microbeads on a magnetic column according to manufacturer’s instructions (Miltenyi, Bergisch Gladbach, Germany). mRNA was extracted from purified T cells and subjected to gene expression analysis using pre-designed Human Immunology Codeset version 2 on nString nCounter (Nanostring Technologies, Seattle, WA). The raw counts were generated using the Human Immunology panel-2 and normalized using endogenous control genes. The change in T cell gene expression levels upon co-culture with Raji cells was calculated by dividing the normalized counts after co-culture with the normalized counts before co-culture to generate after-to-before ratio. A venn diagram of differentially regulated genes in TRuC and CAR-T cells was generated using R-program. Genes normalized to endogenous control were selected based on fold change (greater than two-fold or lesser than 0.5-fold) or *p*-value < 0.05. The heat map was generated for genes with fold change greater than 1.5 or lesser than 0.5 or *p* < 0.05 (Student’s *t*-test). The data analysis was carried out with R-program based nSolver (Nanostring Technologies, Version 3).

### Anti-tumor efficacy in NSG mouse tumor models

All animal studies were approved by the Abpro Preclinical Services and Charles River Laboratories Animal Care and Use Committees. Female NSG mice (NOD.Sg-Prkdc^scid^ Il2rg^tm1Wj^l/SzJ) from the Jackson Laboratory (Bar Harbor, ME), at least 6 weeks old, were used in the studies. For the systemic Nalm6-LUC and Raji-LUC models, 5 × 10^5^ tumor cells were injected into the tail vein 5 days prior to injection of 5 × 10^6^ ε-TRuC-T or CAR-T cells. Tumor cell growth was monitored every 3-4 days using an IVIS imaging system (PerkinElmer, Waltham, MA). Mice that developed hind limb paralysis were euthanized. To establish a subcutaneous xenograft model, 5 × 10^5^ Raji-LUC cells were resuspended in sterile PBS, mixed 1-to-1 with ice cold Matrigel^®^ (Corning, Tewksbury, MA) and then injected subcutaneously in the dorsal hind flank of the mouse. Three days after Raji-LUC injection, effector T cells at 1 × 10^6^ cells per mouse were injected intravenously. For injection of a comparable number of transduced cells in head-to-head comparisons, transduced T cell numbers in the TRuC or CAR-T cell preparations were adjusted by adding non-transduced T cells, if necessary. Tumor growth was monitored as tumor volume with caliper measurement. The volume of tumor was calculated as: Tumor volume = (length × width^2^)/2.

## Supplementary information

Supplementary Information

## Data Availability

The data that support the findings of this study are available from the corresponding author upon reasonable request. Gene expression datasets were deposited with the Gene Expression Omnibus (GEO) data base under the accession number GSE129500.
