# Supplementary material for: Synthetic TRuC receptors engaging the complete T cell receptor for potent anti-tumor response
Source: Nat Commun. 2019 May 7;10:2087. doi: 10.1038/s41467-019-10097-0 (PMC6504948; doi:10.1038/s41467-019-10097-0)
Supplement: Supplementary file 1 — Supplementary Information [file 41467_2019_10097_MOESM1_ESM.docx]

Supplementary information

**Synthetic TRuC Receptors Engaging the Complete T Cell Receptor for Potent Anti-Tumor Response**

Patrick A. Baeuerle^1^, Jian Ding^1^, Ekta Patel^1^, Niko Thorausch^2^, Holly Horton^1^, Jessica Gierut^1^, Irene Scarfo^3^, Rashmi Choudhary^1^, Olga Kiner^1^, Janani Krishnamurthy^1^, Bonnie Le^1^, Anna Morath^2^, Christian Baldeviano^1^, Justin Quinn^1^, Patrick Tavares^1^, Qi Wei^1^, Solly Weiler^1^, Marcela V. Maus^3^, Daniel Getts^1^, Wolfgang W. Schamel^2^, Robert Hofmeister^1^

**Content**

Supplementary Tab.1 Comparisons of TRuCs with conventional CARs

Supplementary Fig. 1 Expression of the TRuCs on Jurkat cells

Supplementary Fig. 2 Most TCRs have incorporated the α-, β-, γ-, and ε-TRuC.

Supplementary Fig. 3 The TRuCs compete with the endogenous corresponding subunits to assemble to TCRs

Supplementary Fig. 4 TRuCs, contrary to CARs are expressed on TCR-positive Jurkat cells, but very little on TCR-negative RPMI-8226 multiple myeloma cells

Supplementary Fig. 5 T cell activation upon binding to CD19+ target cells

Supplementary Fig. 6 Gene expression analysis of TRuC-T and CAR-T cells.

Supplementary Fig. 7 Nalm6 cells do not express ligands for co-stimulatory receptors on T cells

Supplementary Fig 8. Gating strategy for Figure 1c

Supplementary Fig 9. Gating strategy for Figure 3d

Supplementary Fig 10. Gating strategy for Figures 4a and c

| **Investigated Features** | **TRuC-T Cells** | **28ζ CAR-T Cells** | **BBζ CAR-T Cells** | **Observation** |  |
| --- | --- | --- | --- | --- | --- |
| Kill kinetics over 120 h (Figs. 2b and c) | ++ | ++ | ++ | εTRuC-T cells show fastest and most complete kill. Other TRuC-T cells are less active |  |
| Degranulation of T cells (Fig, 2d) | +++ | +++ | +++ | TRuC-T and CAR-T cells have similar effect |  |
| Release of perforin and granzyme A (Figs. 2e and f) | +++ | +++ | ++ | TRuC-T and CAR-T cells have similar effect |  |
| Release of seven different cytokines (Figs. 2g-m) | ++ | +++ | +++ | TRuC-T cells release less cytokines in 5 of 7 cases (which may be beneficial from a safety perspective) |  |
| Anti-tumor activity in s.c. RAJI model tested at three cell doses (Fig. 3a) | +++ | ++ | ++ | Only TRuC-T cells can control s.c. tumor growth at highest cell dose |  |
| Anti-leukemic activity in RAJI model (Fig. 3b) | +++ | + | + | Only TRuC-T cells can control RAJI leukemia |  |
| Anti-leukemic activity in NALM6 model (Fig. 3c) | +++ | + | +++ | TRuC-T cells and BBζ CAR-T cells can equally well control NALM6 leukemia, but not 28ζ CAR-T cells |  |
| Integration of chimeric receptors into TCR complex (Fig. 4) | yes | no | no | Only TRuCs but not CARs can become part of the TCR complex (and thereby leverage its controlled signaling power) |  |
| T cell activation by CD69/CD25 expression (Fig. 5a) | +++ | +++ | +++ | TRuC-T and CAR-T cell show comparable induction of T cell activation markers |  |
| De novo phosphorylation of CD3ε (Fig. 5b) | +++ | - | - | Only TRuC-T cells induce CD3ε phosphorylation of TCR indicative of proper TCR signaling |  |
| De novo phosphorylation of LAT (Fig. 5b) | +++ | + | + | Higher LAT phosphorylation levels seen in TRuC T cells |  |
| Phosphorylation of CD3ζ post expansion phase (Fig. 5c) | - | ++ | ++ | TRuC-T cells show no CD3ζ phosphorylation indicative for absence of tonic signaling |  |

**Supplementary Table 1. Comparisons of TRuCs with conventional CARs.** TRuC-T cell advantage,
 Equivalence of TRuC and CAR-T cells.

**Supplementary Figure 1. Expression of TRuCs on Jurkat cells**

(a) Unmodified Jurkat cells (NT Ctrl) and the stable Jurkat transductants expressing GFP alone (Vector Ctrl) or expressing the TRuCs and GFP or CARs and GFP as indicated were stained with a murine scFv-specific biotinylated anti-F(ab’)2 antibody and APC-conjugated streptavidin as secondary staining reagent. After washing cells were analysed by flow cytometry. The APC fluorescence and GFP intensities are shown. (b) Samples in (a) were analyzed in triplicates. The MFI of the APC fluorescence is shown. Error bars are standard deviation; n=3.

**Supplementary Figure 2. Most TCRs have incorporated the α-, β-, γ-, and ε-TRuC.**

The band intensities of four Blue Native gels as shown in 4a were quantified using ImageQuant TL. The percentage of TCRs on the Jurkat cells with one TRuC (blue) or with two TRuCs (red) is shown. Representative data of three experiments. Samples were measured in quadruplicates.


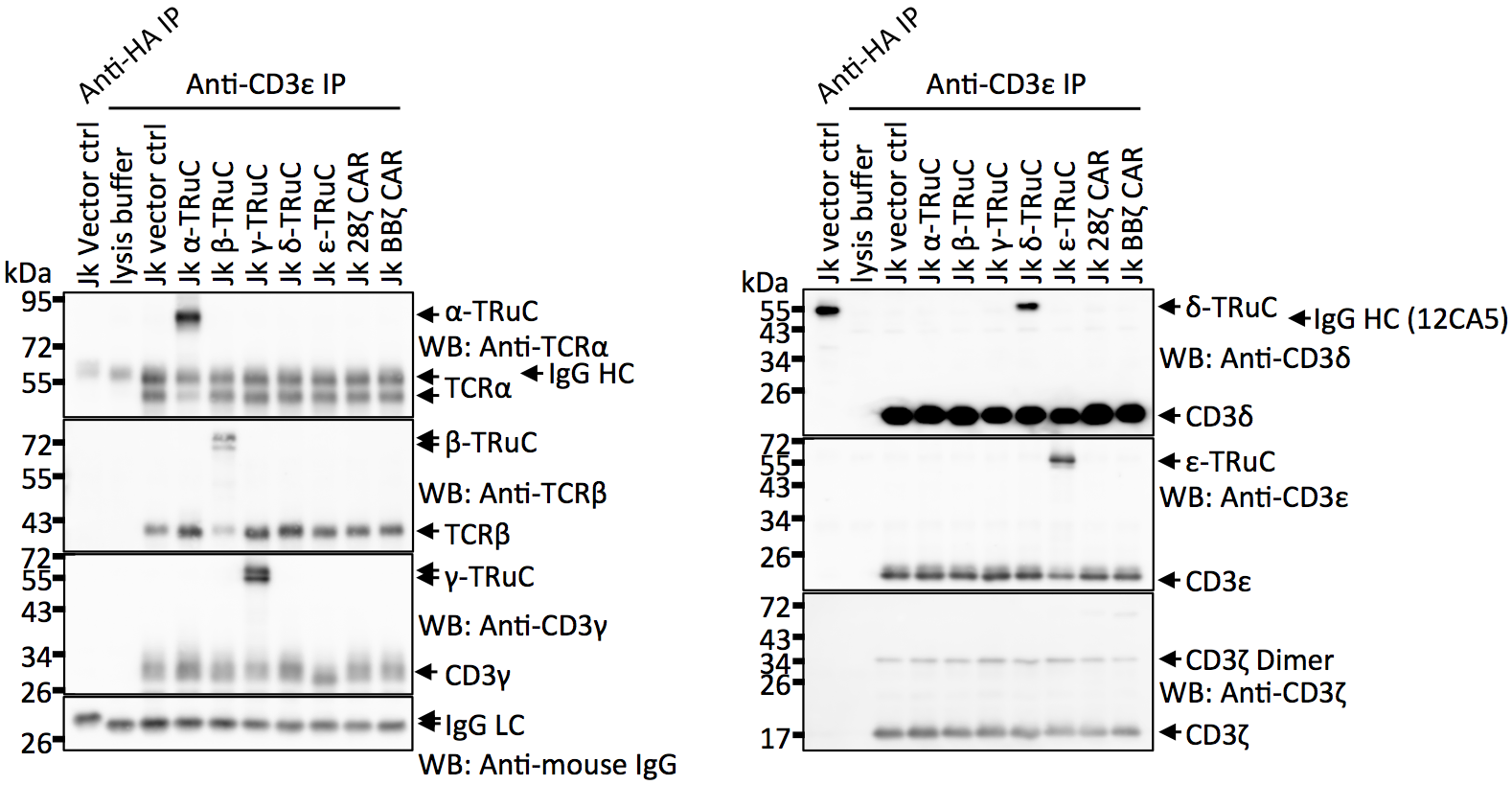


**Supplementary Figure 3. The TRuCs compete with the endogenous corresponding subunits to assemble to TCRs**

Jurkat cells stably transduced with a control vector or vectors encoding for the different TRuCs and CARs were lysed in 0.5% Brij96. CD3 was immunopurified using the anti-CD3ε antibody OKT3 from the cellular lysates, and separated by reducing SDS-PAGE. As a control, the procedure was also applied to the lysis buffer alone and the immunopurification of lysates of Jurkat cells transduced with a control vector was done with the anti-HA tag antibody 12CA5. (Co)-purified proteins were detected by Western blotting using antibodies against each TCR subunit and the light chain (IgG LC) of the anti- CD3ε antibody as a loading control; n=3.

In this experiment all the TRuCs, but not the CARs, were co-purified with CD3, demonstrating again that in contrast to the CARs the TRuCs assemble with the endogenous TCR components. Furthermore, less endogenous TCRα was co-purified with CD3 when the α-TRuC was expressed compared to the vector control or the expression of other TRuCs or CARs. This indicates that the α-TRuC had replaced some of the endogenous TCRα chains, suggesting that the α-TRuC competes with the endogenous TCRα chains for being incorporated into a TCR complex. The same holds true for the β-TRuC, γ-TRuC and ε-TRuC. In case of the δ-TRuC, the reduction in endogenous CD3δ, is not detected, most likely due to the low expression level or low assembly efficiency of the δ-TRuC. Lastly, the endogenous CD3γ chain showed a lower molecular weight when the ε-TRuC was expressed, suggesting that full glycosylation of CD3γ was hindered by the ε-TRuC.

**Supplementary Figure 4. Contrary to CARs, ε-TRuCs are highly expressed on TCR-positive Jurkat cells, but very little on TCR-negative RPMI-8226 multiple myeloma cells**

Surface expression of ε-TRuC and CARs in TCR-positive (Jurkat) and TCR-negative (RPMI 8226) cell lines. The TRuC and CAR constructs were introduced via lentiviral vector into the cells. The transduction efficiency was determined as the percentage of cells expressing GFP. The expression level on the cell surface was assessed by staining with the anti-F(ab’)_2_ antibody. The cells were analyzed using flow cytometry. Representative data of two independent experiments are shown (n=2).

**Supplementary Figure 5. T cell activation upon binding to CD19+ target cells**

The stable Jurkat transductants expressing GFP alone (Vector Ctrl) or expressing the TRuCs and GFP or the CARs and GFP as indicated were co-cultured with CD19+ Raji (a) or CD19+ Daudi (b) tumor cells at a 5:1 effector-to-target ratio for 5 hours. The cells were stained with an APC conjugated anti-CD69 antibody and the amount of CD69 was analyzed by flow cytometry. The graph depicts the MFI of the APC fluorescence in the fraction of GFP positive cells (mean ± SD of triplicates); n=1.

**
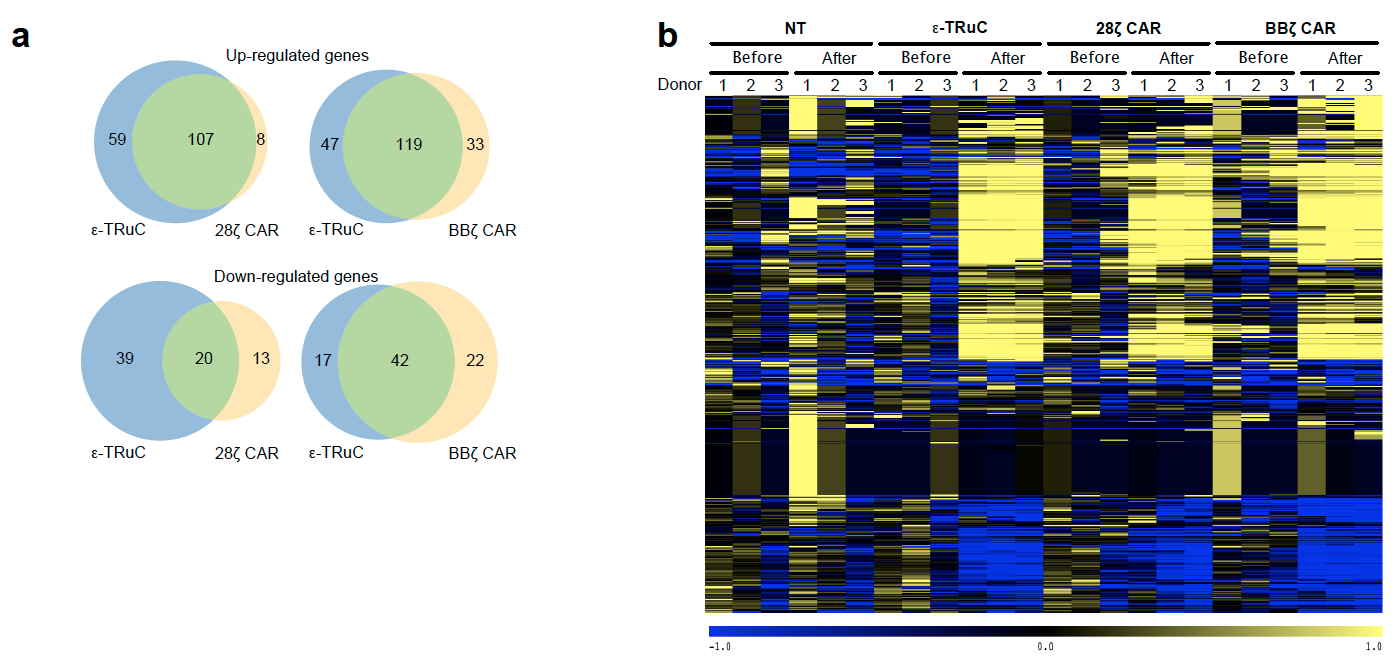
**

**Supplementary Figure 6. Gene expression analysis of TRuC-T and CAR-T cells.**

(a) Venn diagram represents the number of normalized genes that were uniquely regulated in TRuC (shown in blue) or CAR T cells (shown in yellow) or have similar levels of gene regulation in both TRuC- and CAR-T cells (shown in green). (b) Heatmap representing fold change in expression of normalized genes in transduced T cells in comparison to non-transduced (NT) T cells before and after 4-hour tumor cell co-culture. The experiment was carried out once.

**Supplementary Figure. 7. Expression of ligands on Nalm6 and Raji cells for co-stimulatory receptors on T cells**

Raji and Nalm6 cells were surface stained with anti-CD80, anti-CD86, anti-41BBL and anti-ICOS-L antibodies and respective isotype control antibody. Cells were washed and subjected to flow cytometry analysis. Data represents two independent experiments.


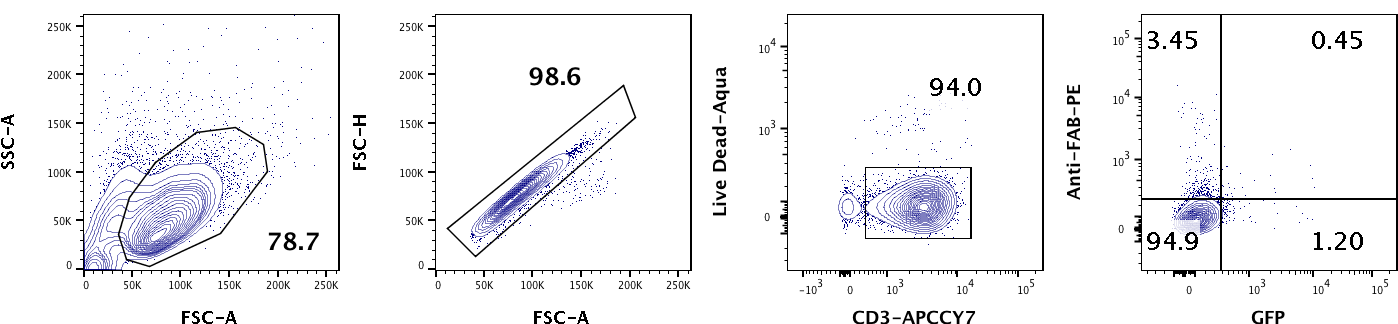


**Supplementary Figure 8. Gating strategy for Figure 1c.**

**Supplementary Figure 9. Gating strategy for Figure 3d**

**
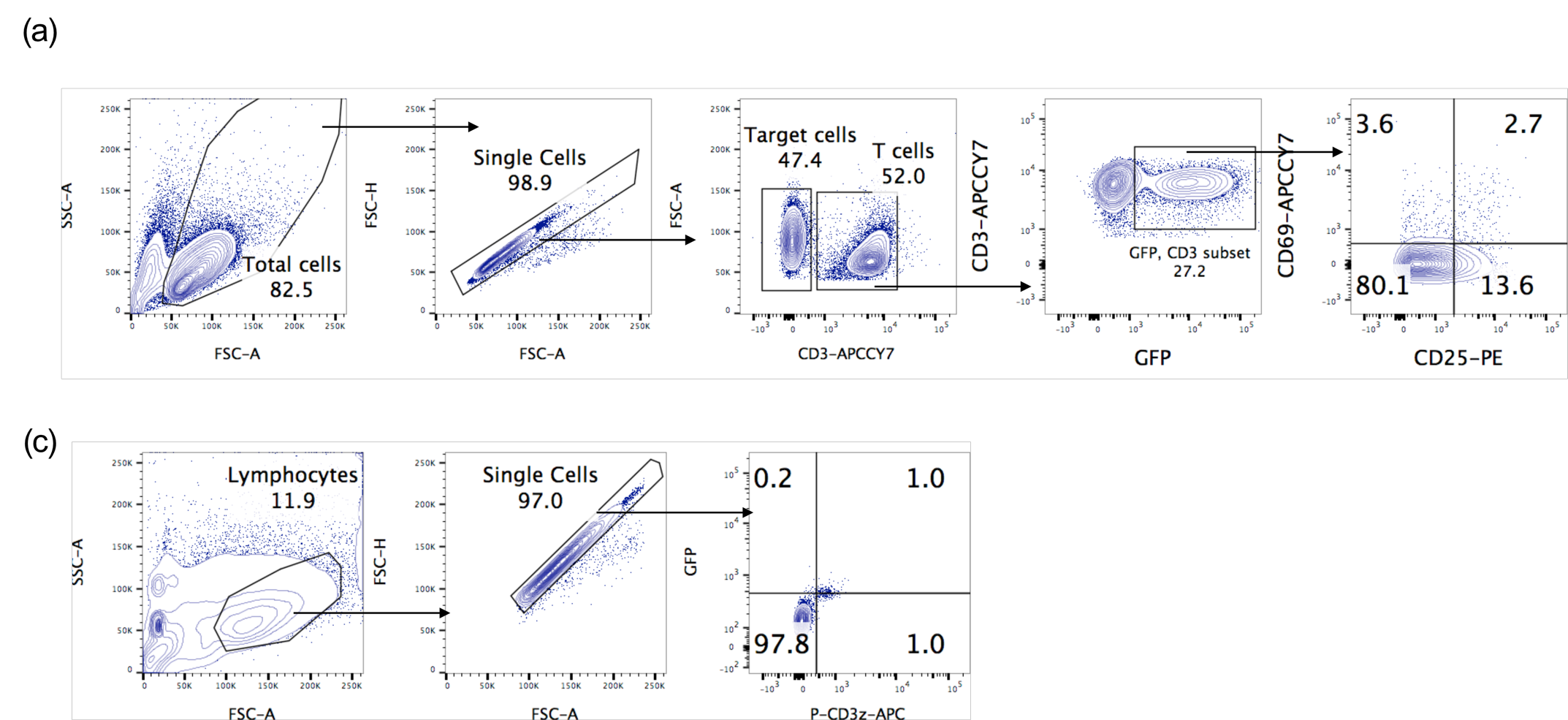
**

**Supplementary Figure 10. Gating strategy for Figures 4a and c**
